# Exercise-induced bronchoconstriction: new evidence in pathogenesis, diagnosis and treatment

**DOI:** 10.1186/s40733-015-0004-4

**Published:** 2015-07-02

**Authors:** Matteo Bonini, Paolo Palange

**Affiliations:** Department of Public Health and Infectious Diseases, “Sapienza” University of Rome, Viale dell’Universita’, 37, 00185 Rome, Italy

**Keywords:** Exercise, Bonchoconstriction, Asthma, Athlete, Sport, Doping, Phenotypes, Biomarkers, Beta-2 agonist

## Abstract

The acute airway narrowing that occurs as a result of exercise is defined exercise-induced bronchoconstriction (EIB). Most recent guidelines recommend distinguishing EIB with underlying clinical asthma (EIB_A_) from the occurrence of bronchial obstruction in subjects without other symptoms and signs of asthma (EIBw_A_). EIB has been in fact reported in up to 90 % of asthmatic patients, reflecting the level of disease control, but it may develop even in subjects without clinical asthma, particularly in children, athletes, patients with atopy or rhinitis and following respiratory infections. Both EIB_A_ and EIBw_A_ have peculiar pathogenic mechanisms, diagnostic criteria and responses to treatment and prevention. The use of biomarkers, proteomic approaches and innovative technological procedures will hopefully contribute to better define peculiar phenotypes and to clarify the role of EIB as risk factor for the development of asthma, as well as an occupational disease.

## Review

### Introduction

The term exercise-induced bronchoconstriction (EIB) describes the acute airway narrowing that occurs as a result of exercise [1]. It has long been known that physical exercise may trigger symptoms of asthma. However, the interest for an objective study on this phenomenon may be dated back to 50 years ago, when Jones and co-workers focused on the physiologic response to exercise in asthmatic children and named the airway obstruction after an exercise challenge exercise-induced asthma (EIA) [2]. Subsequent research defined the different patterns of response to exercise in asthmatic patients, the effect of type, intensity and duration of challenges, and the influence of anti-asthmatic drugs on EIA.

In reviewing these studies, Godfrey concluded that, despite of “some exceptions, there has been no evidence that EIA occurs in patients other than asthmatics, and although sporadic cases have been reported where exercise appears to have been the only precipitant of asthma in a patient (usually an adult), careful investigation has usually revealed other clinical and physiological manifestations of bronchial asthma” [3].

Although some authors consider EIA as a distinct asthma phenotype [4], it is quite evident that exercise may trigger bronchial obstruction and clinical symptoms in almost all asthmatic patients, independently from the causes and mechanisms of the underlying asthma. In fact, the prevalence of EIA has been reported in up to 90 % of asthmatic patients, reflecting the level of disease control, with EIA occurring more frequently in more severe and uncontrolled asthmatic patients [5].

However, the concept that exercise may induce bronchial obstruction in asthmatic patients only is in question [6]. Exercise-induced bronchoconstriction (EIB) may in fact develop even in subjects without clinical asthma. This is particularly true in children, athletes, and patients with atopy or rhinitis, or following respiratory infections [7-10]. It is not easy, however, to report prevalence rates in these populations because they also depend on the type and intensity of exercise, as well as the environmental conditions in which the challenge is performed. Certainly, EIB that occurs in athletes without clinical asthma has peculiar clinical and pathologic features [11] and often disappears after discontinuation of intense training [12].

To bring some clarity to this still controversial issue, a Practice Parameter, jointly developed by the American Academy/College of Allergy Asthma and Immunology (AAAAI/ACAAI) [13], and more recently an American Thoracic Society Clinical Practice Guideline [14], recommended to abandon the term of EIA (because exercise is not the cause, but only a trigger of asthma) and to name EIB with asthma (EIB_A_), the occurrence of bronchial obstruction after exercise in asthmatic patients, and EIB without asthma (EIBw_A_), the occurrence of bronchial obstruction in subjects without other symptoms and signs of clinical asthma.

### Pathogenesis

EIB was initially thought to be secondary to a mediator release from mast cells [3]. This hypothesis was also supported by the refractory period observed after a positive exercise challenge, interpreted as the time needed for mast cell recharge, and by the preventive effect offered by mast cell stabilizing agents, such as sodium cromoglycate. Although mediator release does contribute to cause EIB, pathophysiologic changes induced by intense exercising are definitely more complex. At present, it is widely accepted that hyperventilation through the mouth associated with intense exercise causes the need for humidifying and heating large volumes of air during a short period of time. Elegant experiments performed by S.D. Anderson and coworkers [15] show that the respiratory water loss and the increase in osmolarity of the airways surface liquid represent the major determinants of EIB (osmotic theory). In fact, the dryer and cooler is the inspired air and the higher is the ventilation, the greater is the likelihood of a positive response to exercise, which also explains the higher prevalence of EIB in winter sport disciplines [16]. The vasodilation associated with airways rewarming (thermal theory) may also play a role in inducing bronchial obstruction after exercise [17].

In EIB_A_, the previously mentioned mechanisms only represent a trigger of the underlying airway hyperreactivity in subjects with different phenotypes of asthma not under control.

However, in EIBw_A_, the epithelial damage of a large number of bronchial tree divisions down to peripheral airways represents the predominant pathogenic mechanism [18]. A direct effect of viral infections, occupational agents and exercise may in fact represent a causal mechanism of bronchoconstriction, alternative to the classic eosinophilic mast cell–dependent pathway occurring in allergic asthma [19]. The role of epithelial damage and of substances released by the epithelium and found in sputum, such as interleukin-8 and leukotrienes [20, 21], and in serum or urines, such as Nerve Growth Factor (NGF) and the Clara cell protein CC16 [20, 22], may also explain the heterogeneous inflammatory response reported in EIBw_A_ [23]. The increase of columnar epithelial cells in induced sputum [20] is, in fact, associated with a neutrophilic or mixed eosinophilic–neutrophilic inflammation [24, 25]. The importance of aquaporin, expressed by the subepithelial glandular cells, in regulating the water transport through the epithelium [26], as well as the increased mucus production, as shown by the increased expression of MUC5AC in induced sputum [27], may be also relevant in EIBw_A_.

At last, autonomic dysregulation may also have a role in causing bronchial obstruction in EIBw_A_ [28], both through the basal increased parasympathetic tone shown in athletes and through reflex mechanisms induced by exercise.

### Diagnosis

Research performed over the past twenty years in athletes has consistently revealed a poor relationship between the presence of ‘asthma-like’ symptoms and objective evidence of EIB [29, 30]. Furthermore, pulmonary function tests at baseline appear to be poorly predictive of EIB in athletes, often being within the normal ranges even in the presence of disease [31]. Thus, in order to establish a secure diagnosis of EIB it is important to perform objective testing to confirm dynamic changes in airway function [14].

#### Bronchoprovocative tests (BPTs)

After a careful history and physical examination, measuring the change in the forced expiratory volume in the 1 s (FEV_1_) before and after a standardized exercise challenge test (ECT), in the laboratory or in the field, represents the most intuitive and commonly adopted approach to diagnose EIB [32]. An ECT should be performed in subjects with EIB_A_ only when their baseline FEV_1_ is ≥70 % of normal.

A ≥10 % fall in FEV_1_ at any two consecutive time-point recordings (1, 3, 5, 10, 15, 20, 25, 30 min.) after 6 to 8 min of treadmill or cycloergometer exercise in ambient conditions (20-25 °C; relative humidity <50 %), is considered diagnostic of EIB [32]. The intensity of exercise should be enough to reach in the first 2–3 min. 40-60 % of the predicted maximum voluntary ventilation (estimated as baseline FEV_1_ X 35) or 80-90 % of the predicted maximal heart rate (calculated by 220 - age). Indeed, it has been reported that the mean fall in FEV_1_ after an exercise challenge is more than doubled after achieving 95 % of HR max, compared to 85 % [33]. Standard criteria for laboratory exercise testing may be insufficient to induce a positive response in highly trained individuals and may fail to properly reproduce the bronchoprovocative stimulus experienced by athletes practicing their own sport discipline. Accordingly, sports-specific challenges are also used in athletes. However, these are more difficult to standardize, limiting their application.

Other BPTs may be adopted as surrogate diagnostic tools for EIB. Direct BPTs, (i.e. methacholine and histamine provocation), are accurate to document bronchial hyperreactivity in asthma and in EIB_A_, while indirect tests, such as eucapnic voluntary hyperpnea, hypertonic saline challenge, and mannitol inhaled powder challenge, better reproduce the effects of exercise on the airways and are therefore more accurate to diagnose EIBw_A_ [34]. Though, correlations between exercise tests and other indirect BPTs are at present arguable and vary from test to test [35].

The International Olympic Committee recommends specific thresholds for the various BPTs to document asthma or EIB and to permit the use of anti-asthmatic drugs that are banned or limited by the World Anti-Doping Agency (Table 1).

**Table 1:** International Olympic Committee criteria for the diagnosis of asthma and permission to use of beta-2 agonists: a positive clinical history associated to at least one positive test is required

Diagnostic procedure	Criteria
Pulmonary function	FEV_1_ < 70 %, FEV_1_/VC <55 %
Bronchodilator test	↑ FEV_1_ ≥ 12 % or >200 ml
Eucapnic Voluntary Hyperpnea (EVH)	↓ FEV_1_ ≥ 10 %
Exercise challenge	↓ FEV_1_ ≥ 10 %
Methacholine challenge	↓ FEV_1_ ≥ 20 % with a:
	PC_20_ ≤ 4 mg/ml (for subjects not taking ICS)
	or
	PC_20_ ≤ 16 mg/ml (for subjects taking ICS for at
	least 1 month)
Hyperosmolar test (Mannitol, Saline)	↓ FEV_1_ ≥ 15 %

The differential diagnosis of EIB should take into account physiologic limitations; anxiety; exercise-induced laryngeal dysfunctions, hyperventilation and hypoxemia; dyspnoea on exertion in obese or poorly fit individuals; shortness of breath with exercise due to lung diseases (other than asthma), cardiac diseases; anemia; myopathies [14].

In particular, vocal cord dysfunction and structural glottis abnormalities are increasingly recognized as conditions that may mimic EIB [36]. However, in vocal cord dysfunction, inspiratory stridor during exercise usually resolves within 5 min representing the major differential sign, associated with negative BPT result and poor response to anti-asthmatic drugs. Vocal cord dysfunction may also coexist with EIB.

If pruritus, urticaria or systemic reactions are associated with symptoms of EIB, the diagnosis of exercise-induced urticaria or anaphylaxis should be at last considered [37].

### Treatment and Prevention

Treatment of both EIB_A_ and EIBw_A_ is essentially based on reversing bronchial obstruction by using short-acting beta-2 agonists [5].

#### Pharmacologic Prevention

Several pharmacologic approaches can be adopted to prevent EIB [38]. Because EIB_A_ is a sign of poor asthma control, prevention essentially consists of following international guidelines to achieve asthma control [5]. The potential occurrence of EIB_A_ should not prevent asthmatic patients from an adequate practice of physical exercise, which is not associated to an increased risk of asthma developing or worsening and should instead represent part of their treatment [39]. Multiple therapeutic options may be also appropriate to prevent EIBw_A_, although usually they do not completely avoid the occurrence of bronchoconstriction_,_ but rather attenuate it or shift the dose–response relationship, so that some submaximal efforts become tolerated. Special precautions must be however taken with respect to the World Anti-Doping Agency (WADA) rules on the use of medications for EIB_A_ and EIBw_A_ in competitive athletes (Table 2).

**Table 2:** Most frequently used medications for EIBA and EIBwA and the 2015 World Anti-Doping Agency (WADA) regulations

Treatment	WADA rules	Notes
Antihistamines	Permitted	Second generation molecules should be preferred to avoid side effects
Leukotriene modifiers	Permitted	
Inhaled steroids	Permitted	
Systemic steroids	Prohibited in competition	
Beta-2 agonists	Inhaled Salbutamol (max 1600 mcg/24H), Formoterol (max 54 mcg/24H) and Salmeterol permitted	The presence in urine of salbutamol >1000 ng/mL or formoterol >40 ng/mL is presumed not to be an intended therapeutic use of the substance and will be considered as an Adverse Analytical Finding
	All others prohibited in and out	
	competition	
Mast-cell stabilizers	Permitted	
Anticholinergic agents	Permitted	
Immunotherapy	Permitted	SCIT should not be performed before or after physical exercise

Mast cell stabilizers (not available in all countries), disodium cromoglycate and nedocromil sodium, attenuate both EIB_A_ and EIBw_A_ when inhaled shortly before exercise, but only have a short duration of action [40].

Leukotriene antagonists (i.e. montelukast) have been reported to be effective in preventing EIB_A_ [41]. However, protection may not be complete and occurs in approximately 50 % of subjects [42].

Regular use of inhaled corticosteroid (ICS) represents the therapy of choice for asthma control and therefore is a recommended treatment to prevent EIB_A_ [43]. The prophylactic administration of ICS has been also suggested in EIBw_A_, particularly if physical activity is performed regularly (>3 times per week), representing a repetitive stimulus for the onset of bronchoconstriction [13]. However, the use of ICS in the prevention of EIBw_A_ may be controversial. In fact this pharmacological strategy is at present not supported by ad-hoc designed clinical trials and response to treatment may be impaired in subjects with an underlying neutrophilic inflammatory pattern.

Beta-2 adrenergic drugs, both short- and long-acting (SABA and LABA), when given in a single inhaled dose or with intermittent administration before exercise, are the most effective drugs to prevent both EIB_A_ and EIBw_A_ [44], providing complete protection against exercise (FEV1 fall <10 %) in 68 % of subjects [45]. The effect usually lasts 2 to 4 h for SABA and up to 12 h for LABA. Heterogeneity observed in the efficacy of beta-2 adrenergic agents to prevent EIB is not dependent on the type of molecule used, but rather on the population sample studied, with more variable effects reported in children [46].

However, the chronic use of SABA and LABA often results in a reduction of the duration and/or magnitude of protection against EIB with cross-reacting tolerance to other beta-2 agonists [45, 47]. This loss of efficacy seems to be only partially prevented by ICS [48]. Furthermore, daily use of SABA and LABA may also result in a worsening of EIB [49]. Therefore, SABA and LABA should be used with caution on a daily basis to prevent EIB. At last, LABA administration should be avoided without concomitant use of ICS according to the U.S. Food and Drug Administration (FDA) warning [50].

Ipratropium bromide prevents EIB_A_, although this effect is not consistent among patients and may be variable in the same patient [51]. Whether subjects with EIBw_A_ or with a prevalent autonomic imbalance represent an EIB phenotype more responsive to anticholinergic agents represents an interesting hypothesis, still waiting for further experimental testing [52].

Calcium channel blockers, beta-adrenergic receptor antagonists, inhaled furosemide, heparin, and hyaluronic acid have been studied to prevent EIB with inconsistent results [13].

#### Non-Pharmacologic Prevention

Similar non-pharmacologic preventive measures can be adopted in both EIB_A_ and EIBw_A_ [13]. These include, whenever possible, avoiding exercise in an at-risk air environment because of temperature, humidity, and pollutants or specific allergens in sensitized subjects. Gradual warming-up and cooling-down periods are always suggested. Some athletes also take advantage of the refractory period after bronchial obstruction deliberately induced by hyperventilation or by an intense exercise challenge. The use of face masks which warm and humidify the air has been reported to provide benefits. There is at last some evidence that weight loss and dietary factors, such as a low sodium intake, omega-3 polyunsaturated fatty acids, or a supplementation of ascorbic acid may be helpful in reducing the occurrence and severity of EIB [53].

### Conclusions and Perspectives

Hyperventilation associated with exercise may induce bronchial obstruction in asthmatic patients. However, using the term EIA to define a specific asthma phenotype does not seem appropriate because exercise is only a trigger of a symptom common to all etiologic forms of asthma not under control. EIB may also occur in subjects without clinical asthma. Both EIB_A_ and EIBw_A_ have peculiar pathogenic mechanisms, diagnostic criteria and responses to treatment and prevention. Further research investigations are desirable to better understand relationships and differences between EIB_A_ and EIBw_A_ and to definitely affirm if they represent two different phenotypes. With this regards it is suggested to adopt a scientific approach which includes the use of biomarkers, proteomics and innovative technological procedures [54-57]. Moreover, it should be interesting to assess whether children with EIBw_A_ are at higher risk for developing clinical asthma with age. At last, it should be questioned if EIB in athletes should be considered an occupational disease due to repeated exercising in inadequate environmental conditions [58].
